# Supplementary material for: ADAP1 promotes latent HIV-1 reactivation by selectively tuning KRAS–ERK–AP-1 T cell signaling-transcriptional axis
Source: Nat Commun. 2022 Mar 1;13:1109. doi: 10.1038/s41467-022-28772-0 (PMC8888757; doi:10.1038/s41467-022-28772-0)
Supplement: Supplementary file 1 — Supplementary Information [file 41467_2022_28772_MOESM1_ESM.pdf]

Supplementary Information

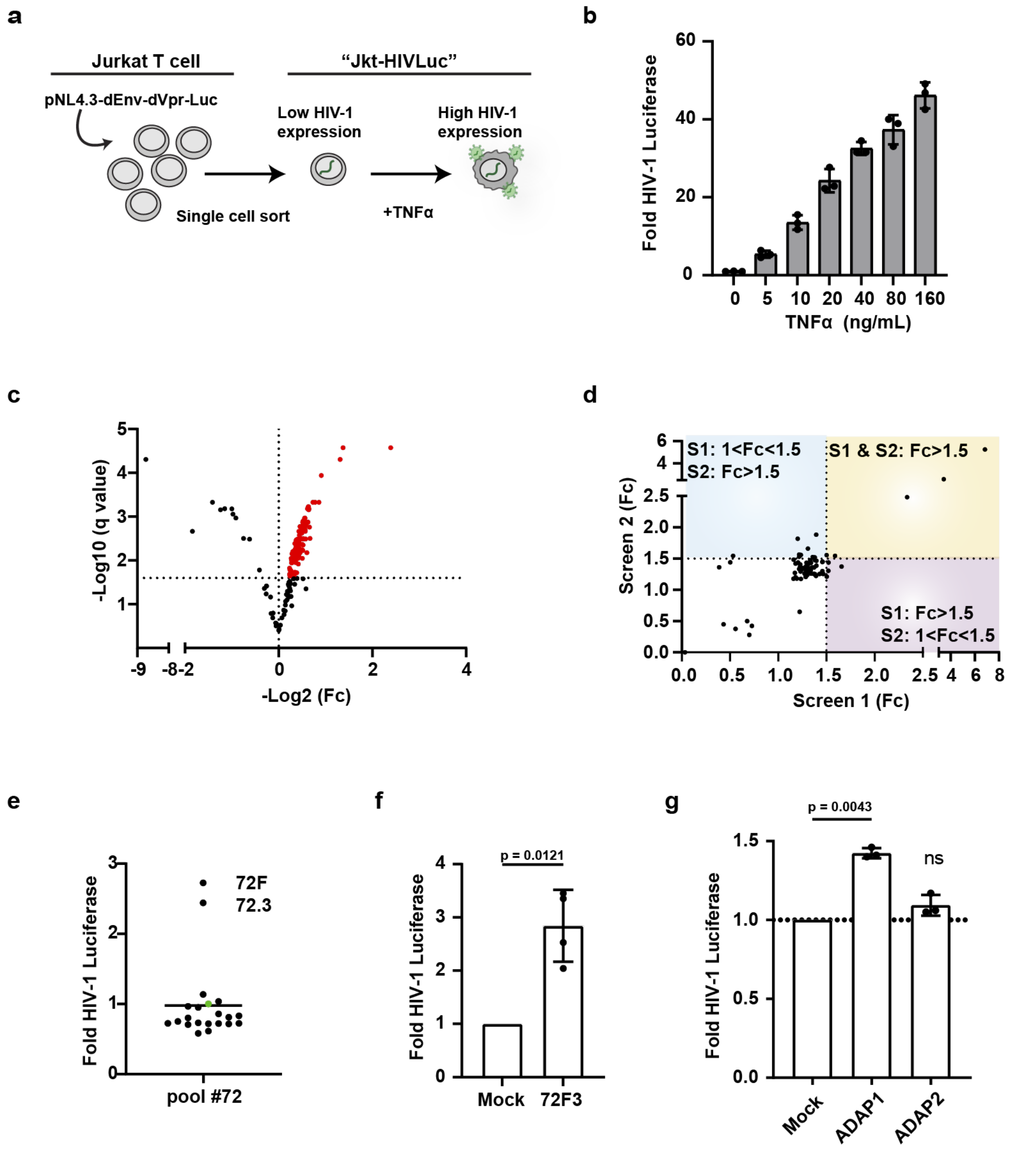

Supplementary Fig. 1. Development of Jkt-HIVLuc, a cell-based model of latency used in the gain-of-function screen to identify undescribed latent HIV-1 activating factors.

a) Schematic of the development of Jkt-HIVLuc cell-based model. Jurkat T cells were transduced with pNL4.3-delta*Env*- delta*Vpr*-Luc-VSVG. Cells were single cell sorted and clonally expanded. Clones were screened by TNF-α treatment (25 ng/mL for 16 hrs) to identify those with latent-reactivation switching capabilities.

b) Selected Jkt-HIVLuc clone responds to TNF-α in a dose-dependent manner exhibiting increasing luciferase activity. Mean ± s.d. fold luciferase activity compared to no treatment (n=3 independent experiments).

c) Volcano plot of second screen to validate first screen (Fig. 1d) using a different batch of generated lentiviral pools. Results are a summary of pooled samples and statistical significance

(determined by multiple unpaired *t* test) for phase 1 gain-of-function screen. Each dot is a pool sample (representing 96 cDNAs) mean luciferase activity (n=3). Dots in red were above the chosen cutoff of $Log_2Fc > 0$ with an $FDR < 2.5\%$.

d) Selection criteria for determining which pools to prioritize for decomposition. Pools with an $FDR < 2.5\%$ and $Log_2Fc > 0$ from both screens (Fig. 1d, Supplementary Fig. 1c) were compared. Pools selected included: the yellow quadrant for pools that induced an HIV-1 Luciferase Fc > 1.5 in both screens; the pink quadrant for pools that induced an HIV-1 Luciferase Fc > 1.5 in screen 1 but still had an Fc > 1 in screen 2; the blue quadrant for pools that induced an HIV-1 Luciferase Fc > 1.5 in screen 2 but still had an Fc > 1 in screen 2.

e) Representative example of screen phase 2 using pool number 72 to identify the activating factor within the pool. Each dot represents the mean ± s.d. fold luciferase activity (n=4) of phase 2 pools (A-H rows= 12 cDNAs each, or 1-12 columns= 8 cDNAs each). The mean activity is represented by the line and the green dot denotes mock control.

f) Representative example of screen phase 3 validating the intersecting well of positive pools from phase 2 (Supplementary Fig. 1e) containing the activating factor. Data represent mean ± s.d. fold of 4 independent experiments (n = 3) normalized to mock. [Paired *t*-test, two-tailed comparing mock and sample independent experiments]. ns= not significant.

g) Fold luciferase activity of Jkt-HIVLuc cells transduced with pTRIP lentiviruses expressing ADAP1 or ADAP2 or mock transduced (no lentivirus). Data represents mean ± s.d. fold luciferase activity (n = 3). [one-way ANOVA followed by Dunnett's test for comparison to mock]. ns= not significant.

Source data are provided as a Source Data file.

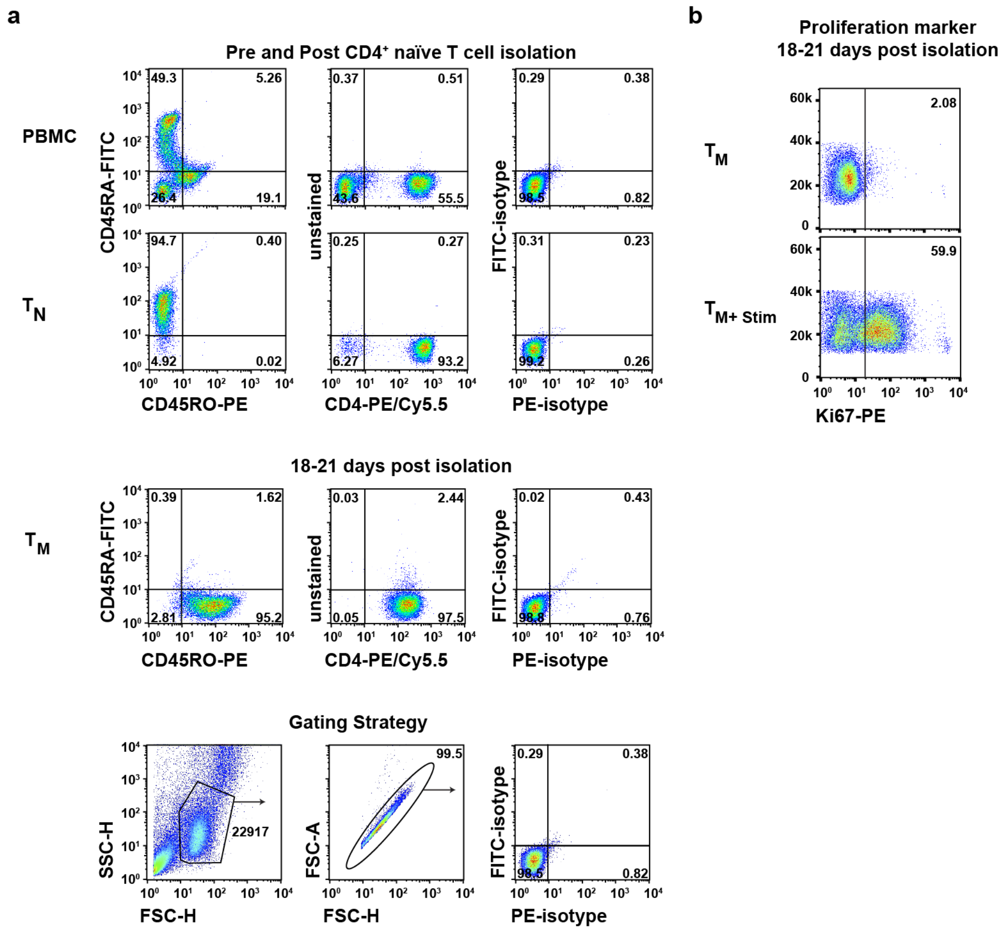

Supplementary Fig. 2. Analysis of primary CD4+ T cell states.

a) Representative flow cytometry analysis comparing purity after naïve CD4+ T cell isolation from PBMC yielding a CD4+CD45RA+CD45RO- population. Approximately 18-21 days post isolation and effector-to-memory transition, population yielded a CD4+CD45RA-CD45RO+ phenotype. Example gating strategy (PBMC sample used) is displayed below, isotype controls were used to set gates.

b) Representative flow cytometric analysis measuring intracellular proliferation marker Ki67 comparing cells that have fully transitioned into resting, non-proliferating memory T (T_M) cells, and stimulated T_M.

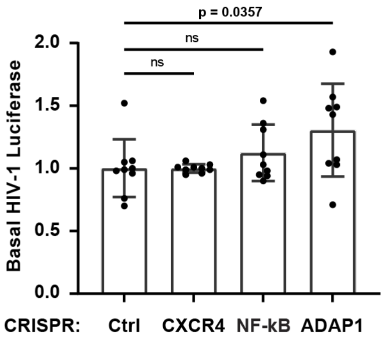

Supplementary Fig. 3. Primary resting ADAP1^{CRISPR} T cells have increased basal HIV-1 activity.

Luciferase analysis of resting HIV-Ctrl^{CRISPR}, HIV-CXCR4^{CRISPR}, HIV-NFκB^{CRISPR} (p65 subunit), and HIV-ADAP1^{CRISPR} T_M cells in the absence of stimulation. For all samples, data represents mean ± s.d. raw luciferase units of 3 donors (n = 3 each) normalized to HIV-Ctrl^{CRISPR} (Ctrl = 1). [one-way ANOVA followed by Dunnett's test for multiple comparison to Ctrl^{CRISPR}]. ns= not significant.
Source data are provided as a Source Data file.

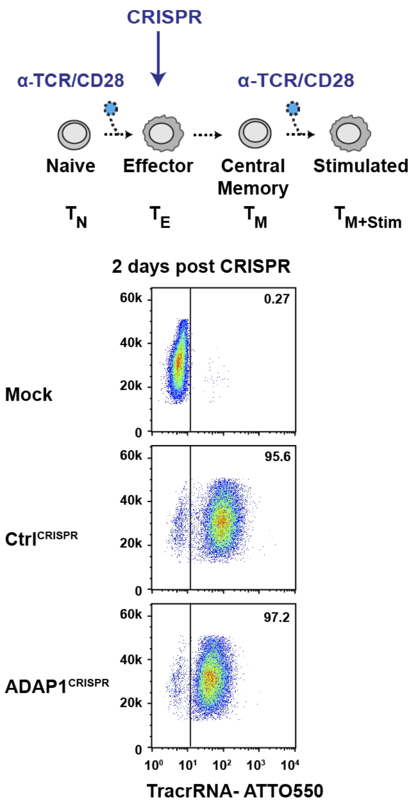

Supplementary Fig. 4. Validating CRISPR-Cas9 ribonucleoprotein electroporation in generation of ADAP1^{CRISPR} and Ctrl^{CRISPR} cells.

Schematic of CRISPR-Cas9 schedule for generating ADAP1^{CRISPR} and Ctrl^{CRISPR} T_M. A ribonucleoprotein (RNP) complex consisting of Cas9 complexed with a fluorescent tracrRNA (ATTO550) and gRNA were assembled *in vitro* before delivering to cells via electroporation. Two days post electroporation, the fluorescent tracrRNA ATTO550 was used to monitor delivery efficiency of the RNP complex by flow cytometry.

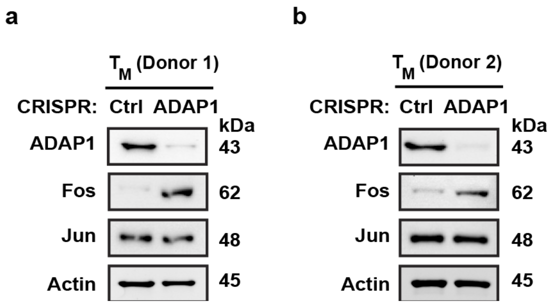

Supplementary Fig. 5. Primary resting ADAP1^{CRISPR} T cells have elevated basal Fos expression.

a) and **b)** Representative western blot analysis of resting Ctrl^{CRISPR} and ADAP1^{CRISPR} T_M in unstimulated donors (n = 3).

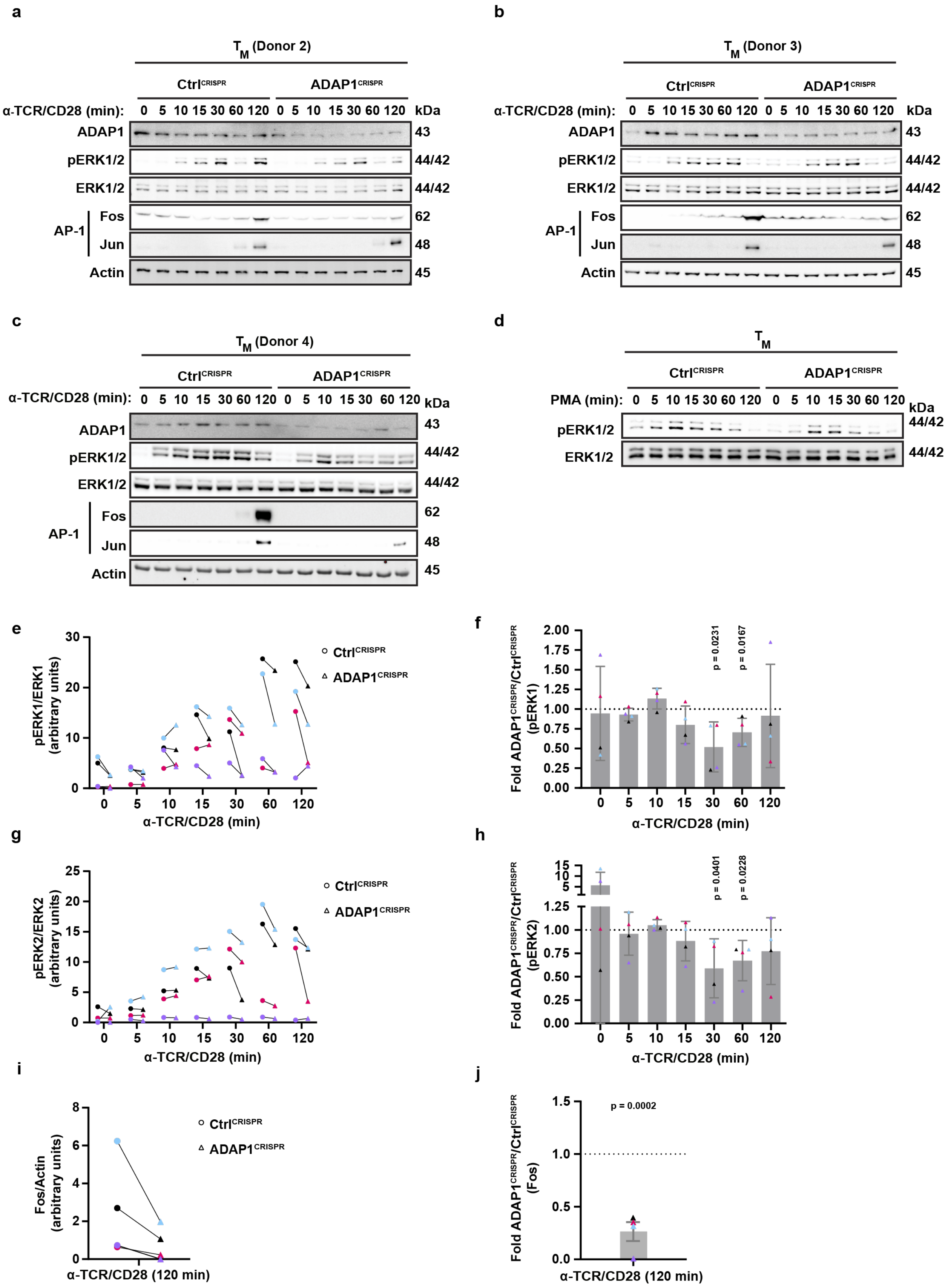
a
T_M (Donor 2)
Ctrl[CRISPR]
ADAP1[CRISPR]
α-TCR/CD28 (min): 0 5 10 15 30 60 120 0 5 10 15 30 60 120 kDa
ADAP1 43
pERK1/2 44/42
ERK1/2 44/42
AP-1 Fos 62
Jun 48
Actin 45
b
T_M (Donor 3)
Ctrl[CRISPR]
ADAP1[CRISPR]
α-TCR/CD28 (min): 0 5 10 15 30 60 120 0 5 10 15 30 60 120 kDa
ADAP1 43
pERK1/2 44/42
ERK1/2 44/42
AP-1 Fos 62
Jun 48
Actin 45
c
T_M (Donor 4)
Ctrl[CRISPR]
ADAP1[CRISPR]
α-TCR/CD28 (min): 0 5 10 15 30 60 120 0 5 10 15 30 60 120 kDa
ADAP1 43
pERK1/2 44/42
ERK1/2 44/42
AP-1 Fos 62
Jun 48
Actin 45
d
T_M
Ctrl[CRISPR]
ADAP1[CRISPR]
PMA (min): 0 5 10 15 30 60 120 0 5 10 15 30 60 120 kDa
pERK1/2 44/42
ERK1/2 44/42
e
pERK1/ERK1 (arbitrary units)
α-TCR/CD28 (min)
Ctrl[CRISPR]
ADAP1[CRISPR]
f
Fold ADAP1[CRISPR]/Ctrl[CRISPR] (pERK1)
p = 0.0231
p = 0.0167
α-TCR/CD28 (min)
g
pERK2/ERK2 (arbitrary units)
α-TCR/CD28 (min)
Ctrl[CRISPR]
ADAP1[CRISPR]
h
Fold ADAP1[CRISPR]/Ctrl[CRISPR] (pERK2)
p = 0.0401
p = 0.0228
α-TCR/CD28 (min)
i
Fos/Actin (arbitrary units)
Ctrl[CRISPR]
ADAP1[CRISPR]
α-TCR/CD28 (120 min)
j
Fold ADAP1[CRISPR]/Ctrl[CRISPR] (Fos)
p = 0.0002
α-TCR/CD28 (120 min)

Supplementary Fig. 6. Loss of *ADAP1* in primary human $CD4^+$ T cells impairs ERK–AP-1 axis activation.

a) **b)** and **c)** Additional donors to complement Fig. 6f. Western blot analysis of primary $ADAP1^{CRISPR}$ and $Ctrl^{CRISPR}$ T_M stimulated with anti-TCR/anti-CD28 beads in a time course-dependent manner from 0 to 120 min (n = 3).

d) Western blot analysis of primary $ADAP1^{CRISPR}$ and $Ctrl^{CRISPR}$ T_M stimulated with 25 ng/mL PMA in a time course-dependent manner from 0 to 120 min (n = 3).

e) Summary of ERK1 phosphorylation normalized to total ERK1 levels in $ADAP1^{CRISPR}$ and $Ctrl^{CRISPR}$ across 4 donors. For panel e-j, donor colors are as follows: donor 1 = black, donor 2 = red, donor 3 = blue, donor 4 = purple. Circles represent $Ctrl^{CRISPR}$. Triangles represent $ADAP1^{CRISPR}$. Donors 1-3 range from 40-73 years old, Donor 4 = 16 years old.

f) Mean ± s.d. fold change of ERK1 phosphorylation normalized to total ERK1 levels in $ADAP1^{CRISPR}$ relative to $Ctrl^{CRISPR}$ in all 4 donors. Dotted line represents Fc = 1 (same Fc in $ADAP1^{CRISPR}$ relative to $Ctrl^{CRISPR}$). [unpaired *t*-test, two-tailed comparing time point to Fc = 1]

g) Summary of ERK2 phosphorylation normalized to total ERK2 levels in $ADAP1^{CRISPR}$ and $Ctrl^{CRISPR}$ across 4 donors.

h) Mean ± s.d. fold change of ERK2 phosphorylation normalized to total ERK2 levels in $ADAP1^{CRISPR}$ relative to $Ctrl^{CRISPR}$ in all 4 donors. Dotted line represents Fc = 1 (same Fc in $ADAP1^{CRISPR}$ relative to $Ctrl^{CRISPR}$). [unpaired *t*-test, two-tailed comparing time point to Fc = 1]

i) Summary of Fos expression normalized to actin levels in $ADAP1^{CRISPR}$ and $Ctrl^{CRISPR}$ across 4 donors.

j) Mean ± s.d. fold change of Fos induction in $ADAP1^{CRISPR}$ relative to $Ctrl^{CRISPR}$ in all 4 donors. Dotted line represents Fc = 1, equal to no difference in Fos levels in $ADAP1^{CRISPR}$ relative to $Ctrl^{CRISPR}$. [unpaired *t*-test, two-tailed comparing time point to Fc = 1].

Source data are provided as a Source Data file.

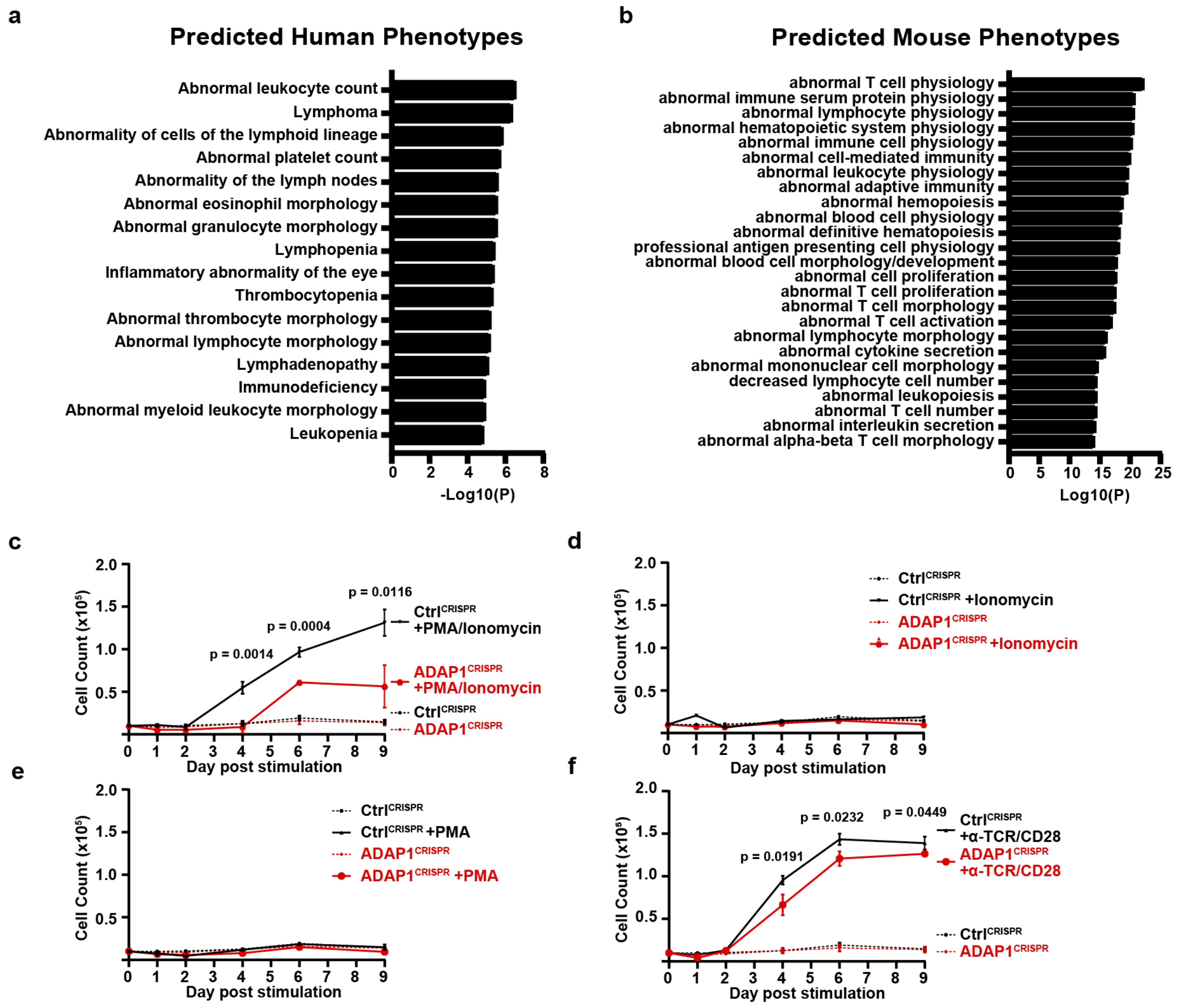

Supplementary Fig. 7. Loss of *ADAP1* predicts immune disease phenotypes and results in reduced T cell proliferation *ex vivo*.

a) Predicted human phenotypes and **b)** mouse phenotypes based on induced genes found in both $ADAP1^{CRISPR}$ and $Ctrl^{CRISPR}$ samples sets. Analysis performed using ToppGene Suites[53].

c) Cell counting analysis of $Ctrl^{CRISPR}$ (black) and $ADAP1^{CRISPR}$ (red) T_M either unstimulated (dashed lines) or stimulated with 25 ng/mL PMA and 1 µM ionomycin (solid lines) in the absence of exogenous growth factor IL2. Data represents mean ± s.d. number of cells (n=3). [unpaired *t*-test, two-tailed between $Ctrl^{CRISPR}$+ PMA/ionomycin and $ADAP1^{CRISPR}$+ PMA/ionomycin].

d) Cell counting analysis of $Ctrl^{CRISPR}$ (black) and $ADAP1^{CRISPR}$ (red) T_M either unstimulated (dashed lines) or stimulated with only 1 µM ionomycin (solid lines) in the absence of exogenous growth factor IL2. Data represents mean ± s.d. number of cells (n=3). [unpaired *t*-test, two-tailed between $Ctrl^{CRISPR}$+ ionomycin and $ADAP1^{CRISPR}$+ ionomycin].

e) Cell counting analysis of $Ctrl^{CRISPR}$ (black) and $ADAP1^{CRISPR}$ (red) T_M either unstimulated (dashed lines) or stimulated with only 25 ng/mL PMA (solid lines) in the absence of exogenous growth factor IL2. Data represents mean ± s.d. number of cells (n=3). [unpaired *t*-test, two-tailed between $Ctrl^{CRISPR}$+ PMA and $ADAP1^{CRISPR}$+ PMA].

f) Cell counting analysis of $Ctrl^{CRISPR}$ (black) and $ADAP1^{CRISPR}$ (red) T_M either unstimulated (dashed lines) or stimulated with anti-TCR/anti-CD28 beads (solid lines) in the absence of exogenous

growth factor IL2. Data represents mean ± s.d. number of cells (n=3). [unpaired *t*-test, two-tailed between Ctrl^{CRISPR}+ anti-TCR/anti-CD28 stimulation and ADAP1^{CRISPR}+ anti-TCR/anti-CD28]. Source data are provided as a Source Data file.

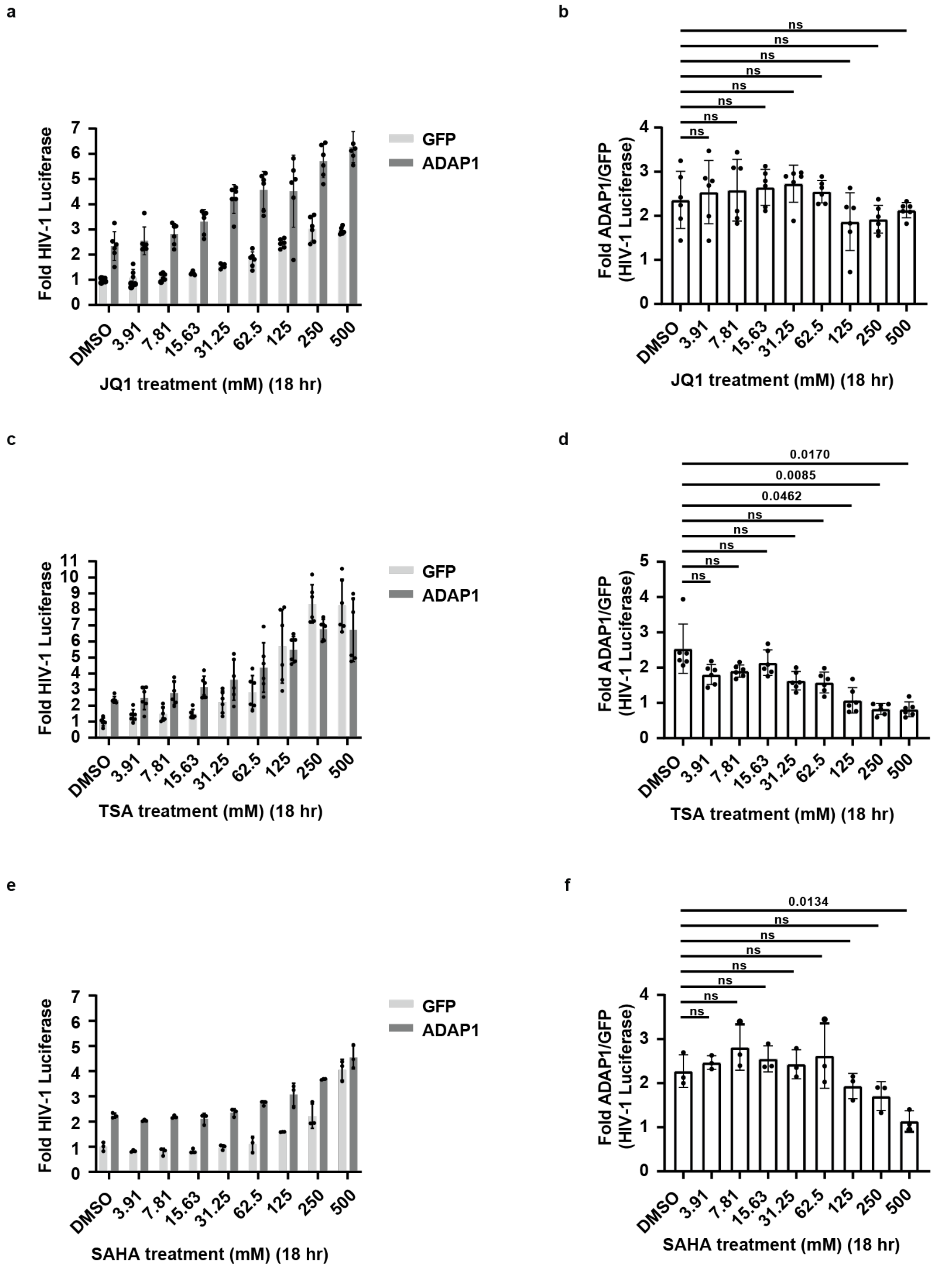

Supplementary Fig. 8. ADAP1 does not work in synergy with latency reversing agents.

a) Fold luciferase activity of Jkt-HIVLuc cells transduced with pTRIP lentiviruses expressing GFP (light gray) or ADAP1 (dark gray) and treated with increasing amounts of JQ1 for 18hrs. Data represent mean ± s.d. fold luciferase activity (n = 6).

b) Mean ± s.d. fold change of HIV-1 luciferase activity in ADAP1 relative to GFP expressing cells treated with increasing JQ1 for 18 hrs (n=6). [one-way ANOVA followed by Dunnett's test for comparison to DMSO]. ns= not significant.

c) Fold luciferase activity of Jkt-HIVLuc cells transduced with pTRIP lentiviruses expressing GFP (light gray) or ADAP1 (dark gray) and treated with increasing amounts of TSA for 18hrs. Data represents mean ± s.d. fold luciferase activity (n = 6).

d) Mean ± s.d. fold change of HIV-1 luciferase activity in ADAP1 relative to GFP expressing cells treated with increasing TSA for 18hrs (n=6). [one-way ANOVA followed by Dunnett's test for comparison to DMSO]. ns= not significant.

e) Fold luciferase activity of Jkt-HIVLuc cells transduced with pTRIP lentiviruses expressing GFP (light gray) or ADAP1 (dark gray) and treated with increasing amounts of SAHA for 18hrs. Data represents mean ± s.d. fold luciferase activity (n = 3).

f) Mean ± s.d. fold change of HIV-1 luciferase activity in ADAP1 relative to GFP expressing cells treated with increasing SAHA for 18hrs (n=3). [one-way ANOVA followed by Dunnett's test for comparison to DMSO]. ns= not significant.

Source data are provided as a Source Data file.

Full western blot images for all figures can be found below.

1g

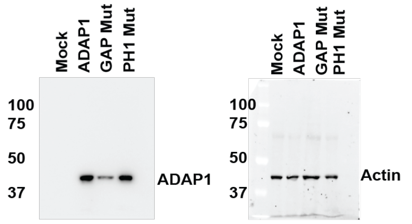

2c

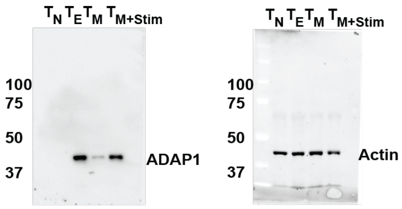

2e

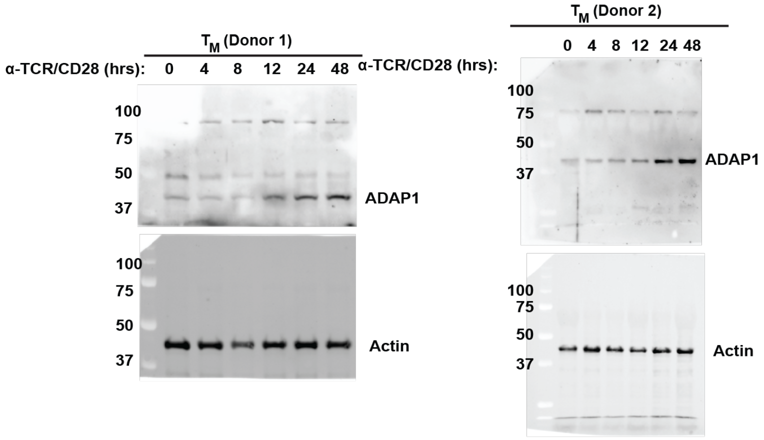

3b

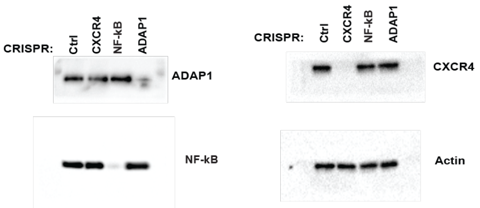

4b

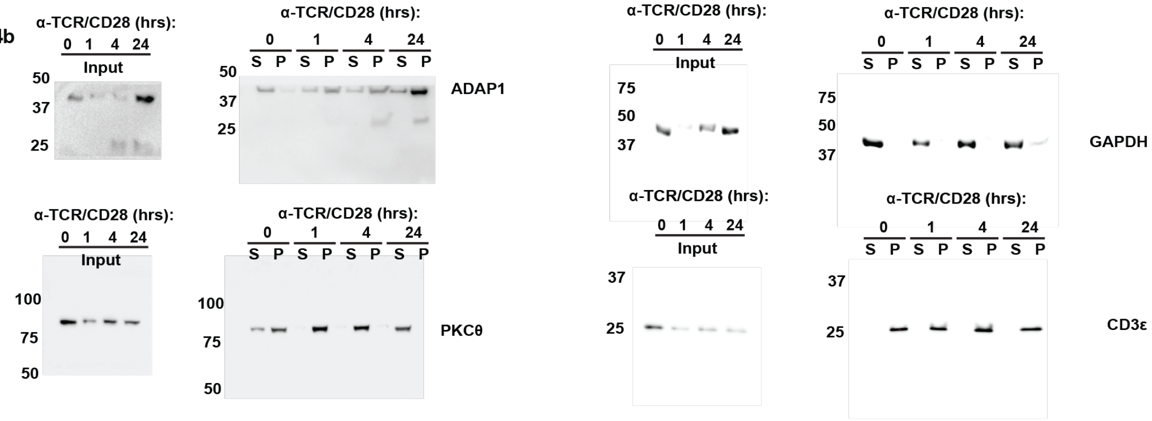

4f

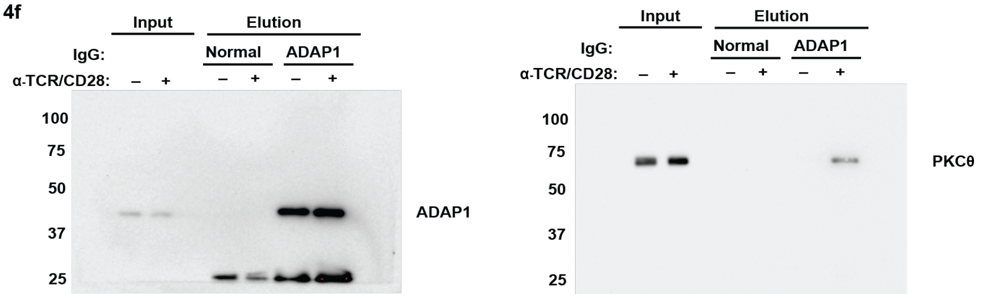

5b

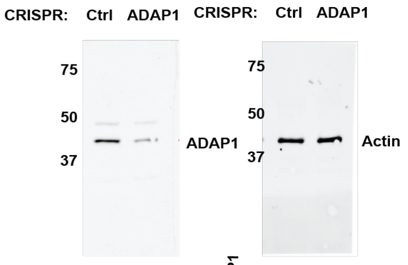

6b

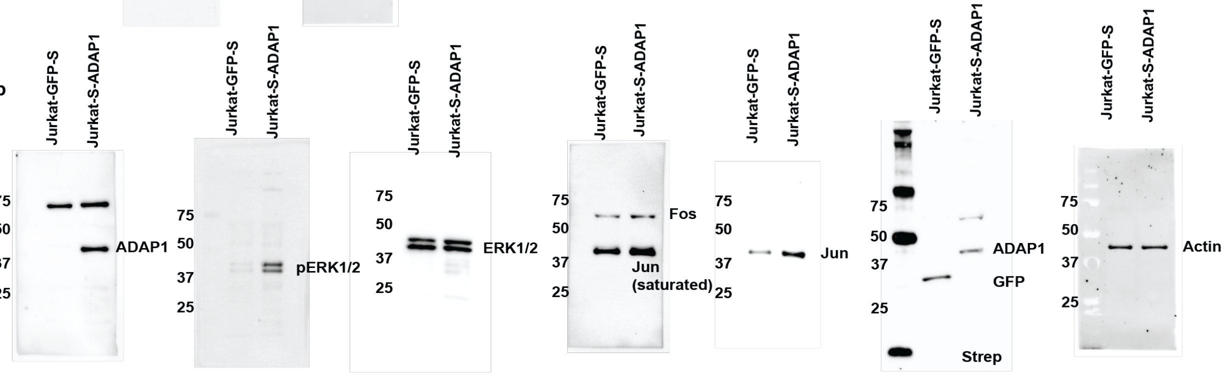

6d

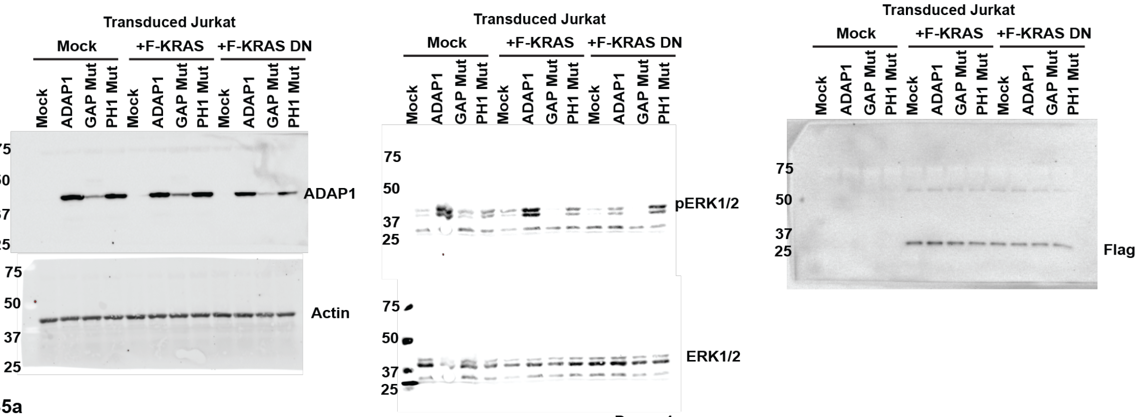

S5a

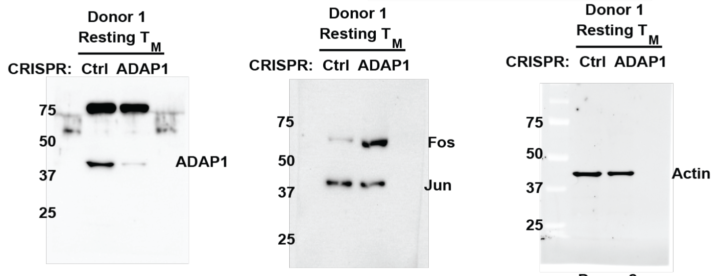

S5b

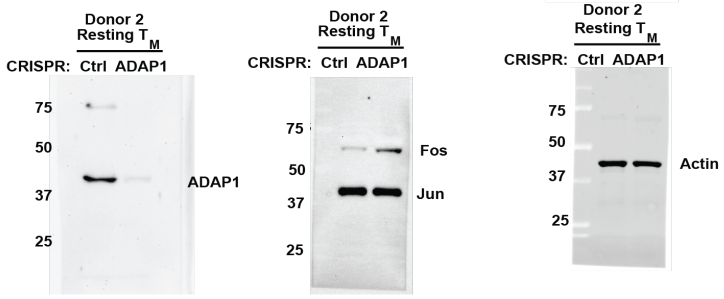

6f

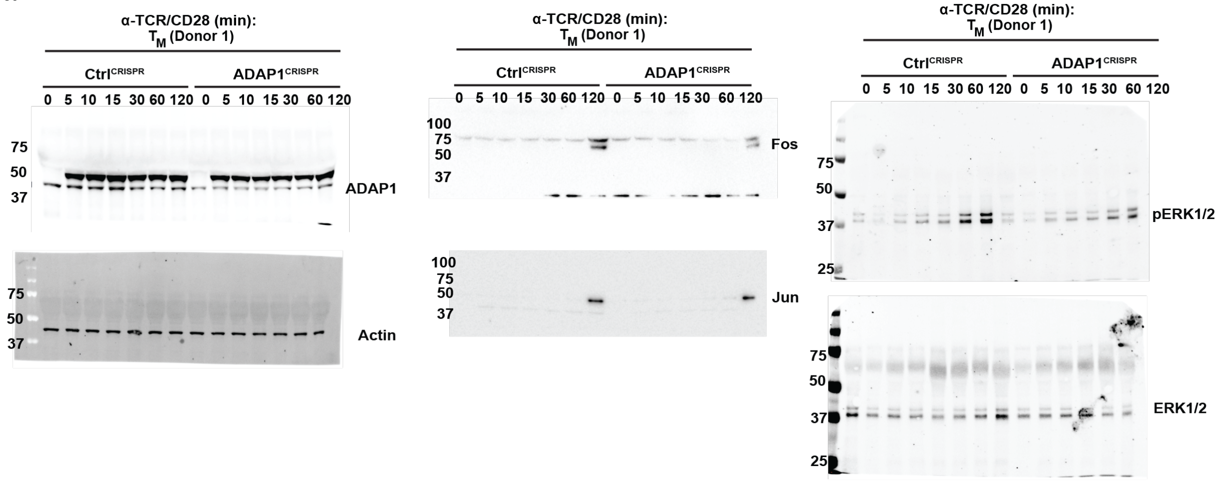
α-TCR/CD28 (min):
T M (Donor 1)
Ctrl CRISPR
ADAP1 CRISPR
0 5 10 15 30 60 120 0 5 10 15 30 60 120
75
50
37
ADAP1
Actin
100
Fos
Jun
pERK1/2
ERK1/2
25

S6a

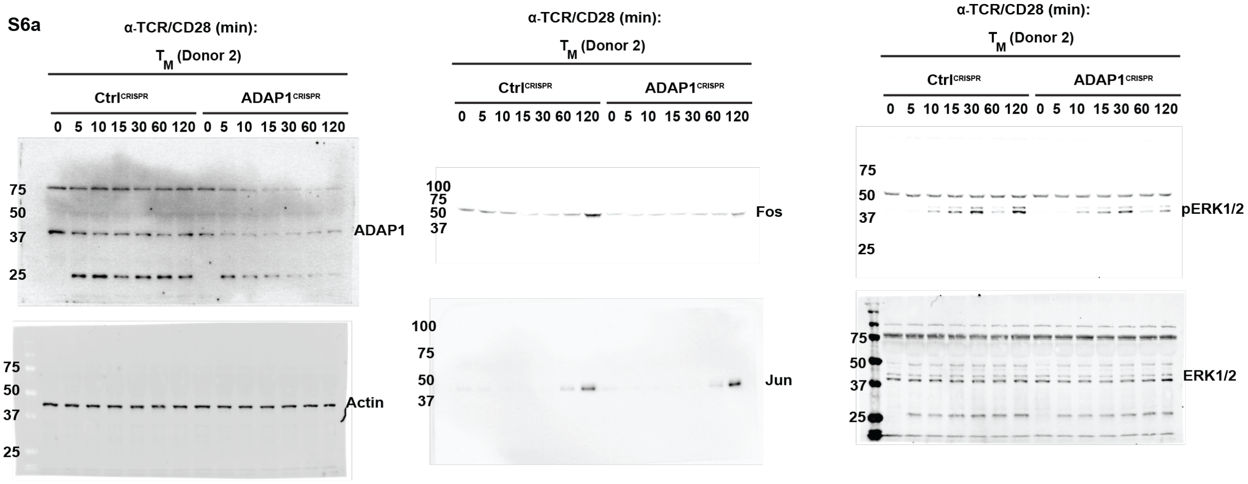
α-TCR/CD28 (min):
T M (Donor 2)
Ctrl CRISPR
ADAP1 CRISPR
0 5 10 15 30 60 120 0 5 10 15 30 60 120
100
75
50
37
25
ADAP1
Actin
Fos
Jun
pERK1/2
ERK1/2

S6b

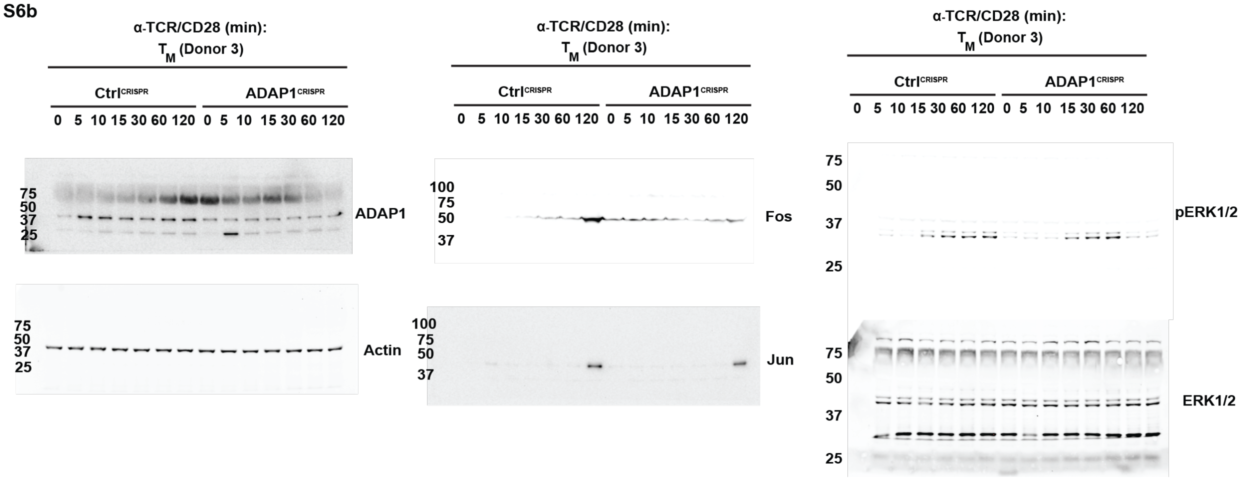
α-TCR/CD28 (min):
T M (Donor 3)
Ctrl CRISPR
ADAP1 CRISPR
0 5 10 15 30 60 120 0 5 10 15 30 60 120
100
75
50
37
25
ADAP1
Actin
Fos
Jun
pERK1/2
ERK1/2

S6c

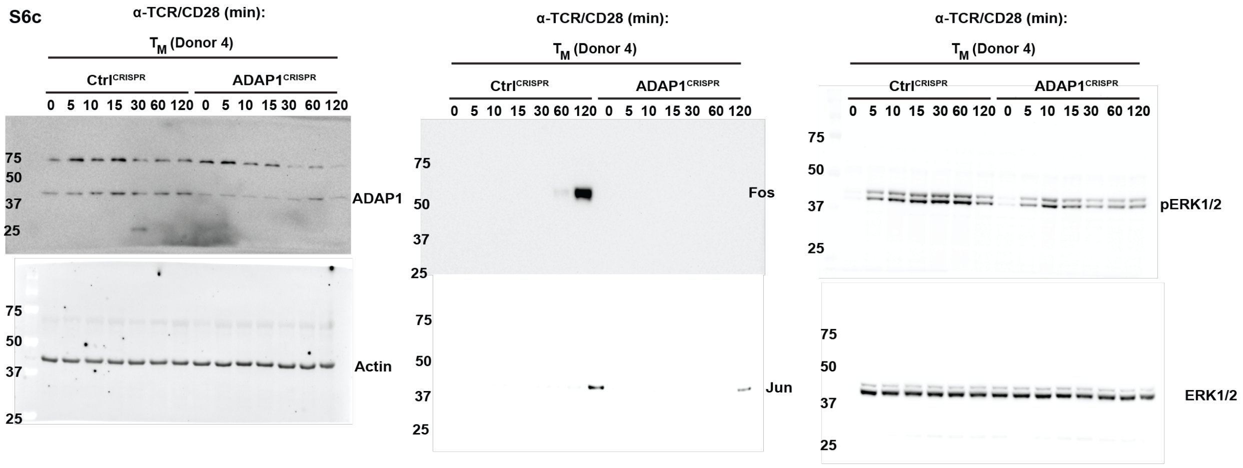

α-TCR/CD28 (min):
T_M (Donor 4)
CtrlCRISPR
ADAP1CRISPR
0 5 10 15 30 60 120 0 5 10 15 30 60 120
75
50
37
25
ADAP1
Actin
Fos
Jun
pERK1/2
ERK1/2

S6d

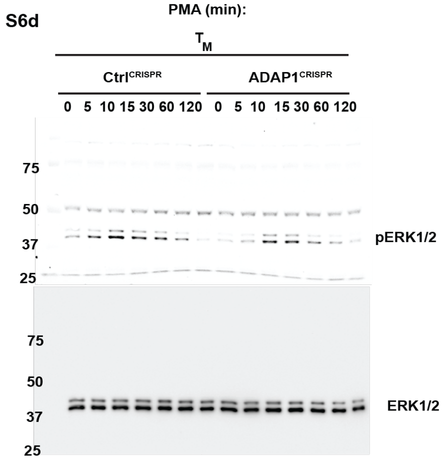

PMA (min):
T_M
CtrlCRISPR
ADAP1CRISPR
0 5 10 15 30 60 120 0 5 10 15 30 60 120
75
50
37
25
pERK1/2
ERK1/2
