# Supplementary material for: ADAP1 promotes latent HIV-1 reactivation by selectively tuning KRAS–ERK–AP-1 T cell signaling-transcriptional axis
Source: Nat Commun. 2022 Mar 1;13:1109. doi: 10.1038/s41467-022-28772-0 (PMC8888757; doi:10.1038/s41467-022-28772-0)
Supplement: Supplementary file 3 — Description of Additional Supplementary Files [file 41467_2022_28772_MOESM3_ESM.pdf]

**Description of Additional Supplementary Files**

File Name: Supplementary Data 1
Description: **ADAP1 IP and mass spectrometry dataset corresponding to donor in Fig. 4b**. File added separately

File Name: Supplementary Data 2
Description: **ADAP1 IP and mass spectrometry dataset corresponding to donor in Fig. 4c-d.** File added separately (

File Name: Supplementary Data 3
Description: **Average Log2Fc of DEG of stimulated vs unstimulated primary T cells corresponding to scRNAseq in Fig. 5c-h.** Two-sided Wilcoxon Rank Sum test. File added separately
