# Supplementary material for: ADAP1 promotes latent HIV-1 reactivation by selectively tuning KRAS–ERK–AP-1 T cell signaling-transcriptional axis
Source: Nat Commun. 2022 Mar 1;13:1109. doi: 10.1038/s41467-022-28772-0 (PMC8888757; doi:10.1038/s41467-022-28772-0)
Supplement: Supplementary file 7 — Reporting Summary [file 41467_2022_28772_MOESM7_ESM.pdf]

nature research

Corresponding author(s): D'Orso, Ivan

Last updated by author(s): 2022.01.05

Reporting Summary

Nature Research wishes to improve the reproducibility of the work that we publish. This form provides structure for consistency and transparency in reporting. For further information on Nature Research policies, see our Editorial Policies and the Editorial Policy Checklist.

Statistics

For all statistical analyses, confirm that the following items are present in the figure legend, table legend, main text, or Methods section.

n/a	Confirmed	
☐	☒	The exact sample size (*n*) for each experimental group/condition, given as a discrete number and unit of measurement
☐	☒	A statement on whether measurements were taken from distinct samples or whether the same sample was measured repeatedly
☐	☒	The statistical test(s) used AND whether they are one- or two-sided<br>*Only common tests should be described solely by name; describe more complex techniques in the Methods section.*
☒	☐	A description of all covariates tested
☐	☒	A description of any assumptions or corrections, such as tests of normality and adjustment for multiple comparisons
☐	☒	A full description of the statistical parameters including central tendency (e.g. means) or other basic estimates (e.g. regression coefficient) AND variation (e.g. standard deviation) or associated estimates of uncertainty (e.g. confidence intervals)
☐	☒	For null hypothesis testing, the test statistic (e.g. *F*, *t*, *r*) with confidence intervals, effect sizes, degrees of freedom and *P* value noted<br>*Give P values as exact values whenever suitable.*
☒	☐	For Bayesian analysis, information on the choice of priors and Markov chain Monte Carlo settings
☒	☐	For hierarchical and complex designs, identification of the appropriate level for tests and full reporting of outcomes
☒	☐	Estimates of effect sizes (e.g. Cohen's *d*, Pearson's *r*), indicating how they were calculated

Our web collection on statistics for biologists contains articles on many of the points above.

Software and code

Policy information about availability of computer code

Data collection	Flow cytommetry: Stratedigm A600 HTAS with software CellCapture v5.0<br>mRNA quality and RT-qPCR assays: 2200 Tapestation (Agilent, 5067-5576) and Applied Biosystems 7500 Fast Real-Time PCR System<br>Immunofluorescence Microscopy: Confocal/Multiphoton Zeiss LSM880 Inverted microscope with 63X oil immersion objective<br>Plate readers: FLUOstar OPTIMA (BMG LABTECH) for luciferase assays and Synergy Neo plate reader (BioTek) for AlphaScreen<br>Western blot images: Chemidoc Imaging system (Bio-rad)<br>Mass Spectrometry: Orbitrap Fusion Lumos mass spectrometer coupled to an Ultimate 3000 RSLC-Nano liquid chromatography system<br>sRNAseq: Countess™ II Automated Cell Counter (ThermoFisher), Qubit 4.0 Fluorimeter (ThermoFisher), Illumina NextSeq500 High Output FlowCell using V2.5 chemistry
Data analysis	Figures and statistical analysis: GraphPad Prism 9.0.2, Adobe illustrator 23.0.01<br>Flow cytometry: FlowJo v10.1<br>Immunofluorescence microscopy: ImageJ 1.53f51/ Java 1.8.0_172 (64 bit) for Windows<br>Western blot analysis: ImageLab v6.0.1<br>scRNAseq analysis: 10X Genomics' analysis pipeline, cellranger (version 3.0.0), STAR (version 2.5.1b), R (version 4.0.2), Seurat (version 4.0.0)<br>Mass Spectrometry analysis: Proteome Discoverer v2.4 SP1 (ThermoFisher Scientific), with peptide identification performed using Sequest HT searching against the human protein database from UniProt<br>Gene ontology and functional interaction analysis: Metascape 3.5 (http://metascape.org), Cytoscape v 3.8.2 (https://cytoscape.org/), String v11.0 (https://string-db.org/)

For manuscripts utilizing custom algorithms or software that are central to the research but not yet described in published literature, software must be made available to editors and reviewers. We strongly encourage code deposition in a community repository (e.g. GitHub). See the Nature Research guidelines for submitting code & software for further information.

Data

Policy information about availability of data

All manuscripts must include a data availability statement. This statement should provide the following information, where applicable:

- Accession codes, unique identifiers, or web links for publicly available datasets
- A list of figures that have associated raw data
- A description of any restrictions on data availability

The scRNA-seq raw and processed sequencing data used in this study are available in the NCBI Gene Expression Omnibus (GEO; https://www.ncbi.nlm.nih.gov/geo/query/acc.cgi?acc=GSE169339) under accession number GSE169339. All other data generated in this study are provided in the main text, Supplementary Information, and Source Data File. Source data are provided with this paper.

Field-specific reporting

Please select the one below that is the best fit for your research. If you are not sure, read the appropriate sections before making your selection.

☒ Life sciences ☐ Behavioural & social sciences ☐ Ecological, evolutionary & environmental sciences

For a reference copy of the document with all sections, see nature.com/documents/nr-reporting-summary-flat.pdf

Life sciences study design

All studies must disclose on these points even when the disclosure is negative.

Sample size	No statistical method was used to predetermine sample size. Sample size was chosen based on experience and published literature (e.g. PMID: 30943398, 34154414, 31175045, 34949807, 34545078) and data provided significant and consistent differences between conditions. For assays involving primary human samples, a minimum of 3 donors were used for each assay (n=2-3 depending on sample material availability per donor).Experiments involving cell lines (Jurkat, Jkt-Luc) were performed a minimal of 3 times. For flow cytometry, at least 20,000 events per sample were collected.
Data exclusions	No data were excluded from analysis
Replication	All in vitro assays were repeated in at least three independent experiments in triplicate. Data generated from primary cells were gathered from multiple independent donors isolated on separate occasions (see legend for number of donors per assay). For lentiviral infections, viral stocks were produced de novo for each independent experiment. All experiments reproduced reliably to support manuscript conclusions.
Randomization	For all in vitro experiments: cells were independently and randomly allocated into groups where they were processed (split, infected, treated, subjected to electroporation, etc) identically at all time points. For assays involving donor cells, each donor served as its own control (i.e. experimental and control samples were generated for each donor (e.g. KO vs Control)) before being subject to treatment (+/- stimulation).
Blinding	Investigators were not blinded during in vitro experiments as all samples were processed and analyzed with the same protocol within an experiment. For scRNA-seq and IP- Mass Spectrometry submitted to core facilities, investigators were blinded to hypothesis, sample treatment.

Reporting for specific materials, systems and methods

We require information from authors about some types of materials, experimental systems and methods used in many studies. Here, indicate whether each material, system or method listed is relevant to your study. If you are not sure if a list item applies to your research, read the appropriate section before selecting a response.

Materials & experimental systems

n/a	Involved in the study	
☐	☒	Antibodies
☐	☒	Eukaryotic cell lines
☒	☐	Palaeontology and archaeology
☒	☐	Animals and other organisms
☐	☒	Human research participants
☒	☐	Clinical data
☒	☐	Dual use research of concern

Methods

n/a	Involved in the study	
☒	☐	ChIP-seq
☐	☒	Flow cytometry
☒	☐	MRI-based neuroimaging

Antibodies

Antibodies used	For western blotting:<br>Anti-Centaurin alpha1 G-4 (ADAP1) (Santa Cruz, sc-390498) (1:1000)

Anti-p65/NF-κB (Santa Cruz, sc-372) (1:4000)
Anti-phos-ERK1/2 Thr202/Tyr204 (Cell Signaling Technology, 4370) (1:1000)
Anti-ERK1/2 (Cell Signaling Technology, 4696 or 4695) (1:2000)
Anti-Fos (Cell Signaling Technology, 2250) (1:1000)
Anti-Jun (Cell Signaling Technology, 9165) (1:1000)
Anti-Flag M2 (Sigma, F31165) (1:10,000)
Anti-PKCtheta (Cell Signaling Technology, 13643) (1:5000)
Anti-CD3ε (Cell Signaling Technology, 4443) (1:5000)
Anti-GAPDH (Cell Signaling Technology, 2118) (1:5000)
hFAB Rhodamine anti-Actin (Bio-Rad, 12004166); (1:10,000)
Anti-StrepTactin-HRP (Bio-Rad, 161-0381) (1:10,000)
Anti-rabbit IRDye 800CW (Licor, 926-32211) (1:10,000)
Anti-mouse IRDye 680CW (Licor, 925-68072) (1:10,000)
Anti-mouse HRP (Cell Signaling Technology, 7076); (1:10,000)
Anti-rabbit HRP (Cell Signaling Technology, 7074), (1:10,000)
Anti-rat HRP (Abcam, ab97057) (1:10,000)

For Flow cytometry:
CD4-PerCP-Cy5.5 (eBioscience, 45-0049-42) (0.125ug/test), control: mouse IgG1k isotype PerCP-Cy5.5 (eBioscience, 45-4714-82) (0.125ug/test);
CD3-PE/Cy7 (BioLegend, 300419) (0.125ug/test), control: mouse IgG1k isotype PE/Cy7 (Invitrogen, 25-4714-80) (0.125ug/test);
CD45RO-PE (eBioscience, 12-0457-41) (0.125ug/test), control: mouse IgG2a isotype PE (eBioscience, 12-4724-42) (0.125ug/test);
CD45RA-FITC (eBioscience, 11-0458-42) (0.125ug/test), CD25-FITC (eBioscience, 11-0257-42) (0.125ug/test), control: mouse IgG2bκ isotype FITC (eBioscience, 11-4732-41) (0.125ug/test).
Ki67 (eBioscience, 12-5699-41) (0.125ug/test), control: mouse IgG1κ Isotype PE (eBioscience, 12-4714-41) (0.125ug/test)

For immunofluorescence and proximity ligation assay:
mouse-anti-ADAP1 (Sigma, SAB1408812)
rabbit-anti-KRAS (Abcam, ab172949)
PLA probes included in Duolink Proximity Ligation Assay (Sigma, DUO92101)
goat anti-mouse IgG H+L Alexa Fluor 594 (Invitrogen, A-11032)

generation of primary human memory T cells:
anti-IL-4 (PeproTech, 500-P24) (1 ug/mL)
anti-IL-12 (PeproTech, P154G) (2 ug/mL)

Validation

Pre-validated and widely used in the literature antibodies were purchased from known reputable manufacturers. Antibodies were additionally validated in over expression, loss of expression systems.

For western blotting:
Anti-Centaurin alpha1 G-4 (ADAP1) (Santa Cruz, sc-390498) https://datasheets.scbt.com/sc-390498.pdf and validated by correct kDa and in over expression and loss of expression system.

Anti-p65/NF-κB (Santa Cruz, sc-372) (1:4000) discontinued, validated by correct kDa and in over expression and loss of expression system.

Anti-phos-ERK1/2 Thr202/Tyr204 (Cell Signaling Technology, 4370) https://www.cellsignal.com/products/primary-antibodies/phospho-p44-42-mapk-erk1-2-thr202-tyr204-d13-14-4e-xp-rabbit-mab/4370 and validated by correct kDa and induction in response to stimulation.

Anti-ERK1/2 (Cell Signaling Technology, 4696 or 4695) https://www.cellsignal.com/products/primary-antibodies/p44-42-mapk-erk1-2-137f5-rabbit-mab/4695?site-search-type=Products&N=4294956287&Ntt=4695%29&fromPage=plp validated by correct kDa.
Anti-Fos (Cell Signaling Technology, 2250) https://www.cellsignal.com/products/primary-antibodies/c-fos-9f6-rabbit-mab/2250?site-search-type=Products&N=4294956287&Ntt=2250%29&fromPage=plp&_requestid=1612208 and
validated by correct kDa and induction upon stimulation.

Anti-Jun (Cell Signaling Technology, 9165) https://www.cellsignal.com/products/primary-antibodies/c-jun-60a8-rabbit-mab/9165?site-search-type=Products&N=4294956287&Ntt=9165%29&fromPage=plp&_requestid=1612275 and validated by correct kDa and induction upon stimulation.

Anti-Flag M2 (Sigma, F31165) https://www.sigmaaldrich.com/US/en/product/sigma/f3165 and validated by correct kDa and over expression system.

Anti-PKCtheta (Cell Signaling Technology, 13643) https://www.cellsignal.com/products/primary-antibodies/pkcq-e1i7y-rabbit-mab/13643?site-search-type=Products&N=4294956287&Ntt=13643%29&fromPage=plp&_requestid=1612309 and validated by correct kDa.

Anti-CD3ε (Cell Signaling Technology, 4443) https://www.cellsignal.com/products/primary-antibodies/cd3e-cd3-12-rat-mab/4443?site-search-type=Products&N=4294956287&Ntt=4443%29&fromPage=plp&_requestid=1612367 and validated by correct kDa and cellular localization.

Anti-GAPDH (Cell Signaling Technology, 2118) https://www.cellsignal.com/products/primary-antibodies/gapdh-14c10-rabbit-mab/2118?site-search-type=Products&N=4294956287&Ntt=2118%29&fromPage=plp&_requestid=1612456 and validated by correct kDa and cellular localization.

hFAB Rhodamine anti-Actin (Bio-Rad, 12004166); https://www.bio-rad.com/en-us/sku/12004166-hfab-rhodamine-anti-tubulin-primary-antibody-40-ul?ID=12004166 and validated by correct kDa .

Anti-rabbit IRDye 800CW (Licor, 926-32211) https://www.licor.com/bio/reagents/irdye-800cw-goat-anti-rabbit-igg-secondary-antibody and validated by correct expected target kDa with minimal background.

Anti-mouse IRDye 680CW (Licor, 925-68072) https://www.licor.com/bio/reagents/irdye-680rd-donkey-anti-mouse-igg-secondary-antibody and validated by correct expected target kDa with minimal background.

Anti-mouse HRP (Cell Signaling Technology, 7076); https://www.cellsignal.com/products/secondary-antibodies/anti-mouse-igg-hrp-linked-antibody/7076?site-search-type=Products&N=4294956287&Ntt=7076%29%3B&fromPage=plp&_requestid=1612524 and validated by correct expected target kDa with minimal background.

Anti-rabbit HRP (Cell Signaling Technology, 7074), https://www.cellsignal.com/products/secondary-antibodies/anti-rabbit-igg-hrp-linked-antibody/7074?site-search-type=Products&N=4294956287&Ntt=7074%29%2C&fromPage=plp&_requestid=1612550 and validated by correct expected target kDa with minimal background.

Anti-rat HRP (Abcam, ab97057) https://www.abcam.com/goat-rat-igg-hl-hrp-ab97057.html and validated by correct expected target kDa with minimal background

For Flow cytometry antibodies listed below, all were validated by comparing to cell staining of cells without markers, cells after bead enrichment and manufacture pre-validation.

CD4-PerCP-Cy5.5 (eBioscience, 45-0049-42), control: mouse IgG1k isotype PerCP-Cy5.5 (eBioscience, 45-4714-82); https://www.thermofisher.com/antibody/product/CD4-Antibody-clone-RPA-T4-Monoclonal/45-0049-42#:~:text=CD4%20Antibody%2C%20PerCP%2DCyanine5.,(45%2D0049%2D42)

CD3-PE/Cy7 (BioLegend, 300419), control: mouse IgG1k isotype PE/Cy7 (Invitrogen, 25-4714-80); https://www.biolegend.com/en-us/search-results/pe-cyanine7-anti-human-cd3-antibody-3070

CD45RO-PE (eBioscience, 12-0457-41), control: mouse IgG2a isotype PE (eBioscience, 12-4724-42); https://www.thermofisher.com/antibody/product/CD45RO-Antibody-clone-UCHL1-Monoclonal/12-0457-42

CD45RA-FITC (eBioscience, 11-0458-42), https://www.thermofisher.com/antibody/product/CD45RA-Antibody-clone-HI100-Monoclonal/11-0458-42

CD25-FITC (eBioscience, 11-0257-42), control: mouse IgG2bκ isotype FITC (eBioscience, 11-4732-41).https://www.thermofisher.com/antibody/product/CD25-Antibody-clone-CD25-4E3-Monoclonal/11-0257-42

Ki67 (eBioscience, 12-5699-41), control: mouse IgG1κ Isotype PE (eBioscience, 12-4714-41) https://www.thermofisher.com/antibody/product/Ki-67-Antibody-clone-20Raj1-Monoclonal/12-5699-41

For immunofluorescence and proximity ligation assay, antibodies validated by over expression systems.
mouse-anti-ADAP1 (Sigma, SAB1408812) https://www.sigmaaldrich.com/US/en/product/sigma/sab1408812

rabbit-anti-KRAS (Abcam, ab172949) https://www.abcam.com/kras-antibody-c-terminal-ab172949.html

PLA probes included in Duolink Proximity Ligation Assay (Sigma, DUO92101)
goat anti-mouse IgG H+L Alexa Fluor 594 (Invitrogen, A-11032) https://www.sigmaaldrich.com/US/en/product/sigma/duo92101

Eukaryotic cell lines

Policy information about cell lines

Cell line source(s)	HEK293T (ATCC, CRL-11268)<br>Jurkat CD4+ T cells (ATCC, TIB-152)<br>Jkt-HIVLuc cells were generated in the D'Orso lab by pNL4.3-deltaEnv- deltaVpr-Luc45 pseudotyped VSV-G lentivirus transduction and single cell sorted by the UTSW Flow Cytometry Core into 96-well plates. Clones were expanded and screened for low basal luciferase activity and high range of inducibility upon TNFα (Sigma, T6674) dose-response stimulation.

Authentication	Purchased cells were authenticated by short tandem repeat (STR) done by ATCC before receiving cells. Jkt-HIVLuc cells were not authenticated, but were controlled for their ability to respond to immune stimulation (TGFb) and their efficiency of lentivirus transduction.
Mycoplasma contamination	all cells were regularly tested for mycoplasm (SouthernBiotech, 13100-01) and were negative throughout the study
Commonly misidentified lines (See ICLAC register)	no misidentified lines present in the study

Human research participants

Policy information about studies involving human research participants

Population characteristics	We did not use human research participants but we purchased sourced leukocytes from Gulf Coast Regional Blood Center. All sourced leukocytes were negative for a wide panel of infectious diseases including HIV. All donors were male and most ranging 40-73 years, with only one young donor age 16. No other information given on report.
Recruitment	All samples were sourced and distributed by Gulf Coast Regional Blood Center randomly. We did not recruit donors, nor have option to select donor characteristics (age, sex, etc) other than requesting negative tests for a panel of infectious diseases. Due to this inability to select samples, bias present in the study include the fact that all samples came from male donors mostly ranging 40-73 years, and only one young donor age 16 whose samples had the highest response to stimulation.
Ethics oversight	N/A

Note that full information on the approval of the study protocol must also be provided in the manuscript.

Flow Cytometry

Plots

Confirm that:

- [x] The axis labels state the marker and fluorochrome used (e.g. CD4-FITC).
- [x] The axis scales are clearly visible. Include numbers along axes only for bottom left plot of group (a 'group' is an analysis of identical markers).
- [x] All plots are contour plots with outliers or pseudocolor plots.
- [x] A numerical value for number of cells or percentage (with statistics) is provided.

Methodology

Sample preparation	All samples were prepared by transferring 2x105 cells per sample to an uncoated V-bottom 96-well plate (Nunc). The samples were spun down at 300 g for 5 min at room temperature and washed with 0.2 mL 1X PBS twice. Cells were stained according to target protein. Samples were run on a 96-well plate reader (A600 HTAS, Stratedigm) with a gate cell count set at 20,000 events using CellCapTure flow cytometry software (Stratedigm). Data analysis was performed with FlowJo version 10.1.<br>For the detection of RFP, cells were fixed with 1% paraformaldehyde (PFA) for 5 min at room temperature. The PFA was washed twice with 0.2 mL of 1X PBS before resuspending in 0.1 mL 1X PBS/2% FBS and immediately analyzing by flow cytometry.<br>For the detection of cell surface proteins, cells were resuspended in 0.1 mL staining buffer (1X PBS/2% FBS) and incubated for 20 min on ice. Cells were then stained with antibodies against target or appropriate isotype control according to manufacturer's protocol and incubated on ice protected from light for 30 min. After incubation, cells were washed twice in staining buffer, resuspended in 0.15 mL staining buffer, fixed as stated above and analyzed immediately by flow cytometry.<br>For the detection of intracellular proteins, cells were fixed as stated above and permeabilized using 1X BD Perm/Wash (554723) for 15 min at room temperature and then stained with appropriate antibody or isotype control and incubated for 30 min at 4°C protected from light. Cells were washed twice with 1X BD Perm/Wash before being resuspended in staining buffer and were immediately analyzed by flow cytometry.
Instrument	Stratedigm A600 HTAS with software CellCapture v5.0
Software	CellCapture v5.0 and FlowJo v10.1
Cell population abundance	no sorting was involved in the study
Gating strategy	For all Flow cytometry analysis, population corresponding to viable lymphocytes were gated on a FCS/SSC, of which there would have been a minimum 20,000 events collected. Next, singlets were gated using FCS-H/FCS-S gating. Lastly, isotype control staining were used to set positive/negative gates. Example gating strategy in Supplementary Fig 2a.

- [x] Tick this box to confirm that a figure exemplifying the gating strategy is provided in the Supplementary Information.
